# EpiF*AMU*: an epidemiological tool for monitoring farm-level factors essential to antimicrobial use surveillance and infection prevention and control programs in poultry production systems in Zambia—development and application

**DOI:** 10.3389/fvets.2026.1963471

**Published:** 2026-10-22

**Authors:** Isaac Silwamba, Alina Kirse, Geoffrey Mainda, Ntombi Mudenda, Niwael Jesse Mtui-Malamsha, Maria Hartmann, Sandra Brogden, John Bwalya Muma, Lothar Kreienbrock

**Affiliations:** 1 Department of Disease Control, School of Veterinary Medicine, University of Zambia, Lusaka, Zambia; 2 Institute of Biometry, Epidemiology, and Information Processing, University of Veterinary Medicine Hannover, Hannover, Germany; 3 Food and Agriculture Organization of the United Nations, Lusaka, Zambia; 4 Department of Clinical Studies, School of Veterinary Medicine, University of Zambia, Lusaka, Zambia

**Keywords:** antimicrobial resistance, antimicrobial stewardship, critically important antibiotics, treatment frequency, VetCAb-ID

## Abstract

In recent years, Zambia and other countries have intensified their efforts to combat antimicrobial resistance (AMR). In the animal health sector, farm characteristics significantly affect AMR dynamics through antimicrobial use (AMU). In this regard, monitoring these farm factors remains essential for providing insights into the relation between AMU and AMR. Previously, we have shown the feasibility of monitoring AMU at the farm level using the Veterinary Consumption of Antibiotics - International Documentation (VetCAb-ID) system (©TiHo Hannover, Germany). In this study, we describe the development and application of EpiF*AMU*, a tool designed to monitor factors essential to AMU and infection prevention and control at the farm level. This epidemiological tool can monitor a series of distinct epidemiological variables across 13 key domains of poultry management, namely nutrition, vaccination, biosecurity, brooding, housing, equipment, personnel, market practices, extension services, AMR, AMU, mortality rates, and public health. We utilized EpiF*AMU* to assess AMU factor patterns in 45 broiler chicken farms in the Lusaka district of Zambia. Using the Wilcoxon test, an analysis of treatment frequency per day and farm factors revealed statistical significance for six variables: the belief that maximum production cannot be achieved without antimicrobials (*p* = 0.0393), the number of drinking water dispensing points (*p* = 0.0242), vitamin supplementation (*p* = 0.0328), vitamin administration prior to vaccination (*p* = 0.0094), measures to improve chick survival (*p* = 0.0324), and litter thickness (*p* = 0.035). Our findings demonstrate the feasibility of monitoring farm factors alongside AMU monitoring programs to generate valuable insights into potential risk factors within poultry farms that may lead to the use of antimicrobials.

## 1 Introduction

Antimicrobial resistance (AMR) is one of the major public health issues today, threatening the effective prevention and treatment of an ever-increasing range of infections caused by bacteria, parasites, viruses, and fungi. In recent years, Zambia and other countries have intensified their efforts to combat AMR. However, farm factors such as management practices shape patterns of antimicrobial use (AMU), which in turn influence the development and spread of AMR (1–5). It is well known that over 60% of all antibiotics produced globally are used in animal production for both therapeutic and non-therapeutic purposes (6). Many farmers, especially those in resource-constrained settings, face challenges related to poor or inadequate farm-level management practices, limited knowledge, and a high disease burden with insufficient infection prevention and control (IPC). These factors contribute to imprudent AMU and the emergence and spread of AMR (3, 7, 8). Consequently, monitoring farm factors associated with AMU is essential to managing AMR by enabling evidence-based stewardship, enhancing farm management, directing policy, and safeguarding animal and public health (9–11). However, the majority of governments in resource-limited countries lack a robust system to capture AMU data at the farm level due to resource constraints and implementation challenges. Therefore, the use of proxy indicators could provide a suitable alternative.

Poultry is one of the most consumed sources of animal protein in Zambia and many countries worldwide, but it is also a sector that uses large amounts of antimicrobials during production, which raises the risk of AMR (6). Good poultry farming practices are critical and include providing a carefully controlled environment that ensures the comfort of the birds. Overall, to maximize productivity and profitability, poultry farming integrates various factors, including health management, biosecurity, housing, feeding, and environmental control (12, 13). Some relationships between various farm-level factors and the emergence and spread of AMR in poultry production have been reported. For example, the type of production system, such as the intensive management systems used on many poultry farms, has been associated with an increase in AMR due to high animal density and frequent use of antimicrobials (14). Farm size and the presence of different animal species on the same farm have been reported as risk factors for the emergence and spread of resistant bacteria (15, 16). Inadequate biosecurity procedures, such as failing to clean equipment or disposing of dead birds properly, have been linked to higher levels of AMR (17). Farms with fish ponds have been identified to create potentially AMR-promoting conditions, possibly due to cross-contamination and shared water sources (18).

Furthermore, farmer characteristics such as gender, marital status, educational status, and level of awareness regarding resistance have been related to AMR (19–21). Some characteristics of the chickens themselves, such as their age, sex, and breed, have also been shown to influence their susceptibility to diseases that necessitate frequent treatment, which in turn affects the development of AMR (22). Feed type, drinking water source, and proximity of farms to one another have also been associated with increased AMR risks (23, 24). Additional factors contributing to the complexity of AMR include the cleanliness of containers used for medical treatment, the absence of drinking water treatment, frequent feed changes during production cycles, the types of litter material used in chicken hatcheries, the drainage systems in poultry houses, and the lack of footbaths (19, 21, 24, 25).

Similarly, several studies have also reported significant relationships between various farm-level factors and AMU, identifying it as the main driver of AMR. Factors such as farm size, the presence of other livestock species, the intrusion of wild birds into poultry houses, easy access to antibiotics, a high disease burden, and economic constraints have all been associated with increased AMU (26–29). Vaccination procedures, the use of disinfectants for cleaning, the presence of rodents in poultry houses, biosecurity measures, dosing practices, types of poultry management systems used, and socio-financial situations of poultry farmers are some additional factors affecting AMU (30–33). The gender of the farmer, their professional training in farming, educational status, understanding of antimicrobial usage guidelines, compliance with withdrawal periods, farm location, types of production systems, years of farming experience, average number of birds kept, the use of all-in-all-out farming systems, and proximity to veterinary and agro-veterinary pharmacies have all been reported to influence the behaviors and patterns of AMU (34–37).

Despite the importance of these relationships between farm factors, AMU, and AMR, substantial gaps exist in the collection and monitoring of data on farm factors that impact AMR and AMU. Data collection on farm factors is often limited by the lack of standardized monitoring tools and protocols, making it difficult to benchmark farms and to fully understand how specific practices influence AMU patterns and AMR development. There is considerable variation in farm factors across different farms, which suggests that existing protocols, primarily based on traditional questionnaires, may not be sufficient to capture the diversity of farm management practices or the psychosocial factors influencing farmer behavior (9, 38). Moreover, traditional questionnaires are inconsistent, lack harmonization, generate fragmented data, and are mostly unvalidated, making it difficult to monitor farm parameters across geographies and livestock systems consistently and challenging to compare data or evaluate the efficacy of interventions (39, 40). As AMU on farms is influenced by a multifaceted set of factors, standardization of protocols is invaluable for developing interventions that promote responsible use of antimicrobials and reduce the emergence of AMR. Standardization of protocols may also allow objective evaluation of the level of compliance with antimicrobial stewardship (AMS) program requirements and IPC strategies.

In this study, we expand on our previous work on the quantitative analysis of AMU in Zambian poultry production systems (41) by developing a comprehensive epidemiological procedure for monitoring farm-level factors. Our hypothesis was that the EpiF*AMU* tool would demonstrate acceptable internal structure and make it possible to monitor farm-level variables that are essential to AMU and IPC programs in broiler production systems in Zambia. The primary objectives of this work were to develop a comprehensive and contextually relevant tool that is tailored to common poultry farming practices, validate the tool using statistical testing and conceptual models to ensure its applicability and reliability, and then use the validated tool to assess the relationship between specific farm-level factors and the quantitative use of antimicrobials in small- to medium-sized broiler flocks in Zambia. This is particularly relevant in the light of the current global efforts to combat AMR.

## 2 Materials and methods

This study focuses on AMU in poultry in Zambia and the development and application of a tool for AMS programs in poultry production within the country. Therefore, the tool is described here in a multi-step approach. Ethical approval for the study was sought from Excellence in Research Ethics and Science (ERES), Ref. No. May-021.

### 2.1 Development of the tool

#### 2.1.1 Primary questionnaire and EpiF*AMU* documentation tool

The “EpiF*AMU*” tool used here was developed from the primary questionnaire, which was designed as part of this study using a 9-point framework by the Food and Agriculture Organization of the United Nations (FAO), with some modifications made to transition from length-checking to burden-tracking (Modifying Step 7) (42). Following a thorough desk analysis of common practices in poultry production systems, the data needed for the questionnaire were determined. Owners, managers, or supervisors of farms were the intended responders. In-person farm visits were conducted as the means of reaching these target responders. A variety of closed-ended, open-ended, and open-response-option questions spanning a wide range of practices in poultry production systems were used in the development and organized in 13 domains that are potentially related to animal health. These domains were nutrition, veterinary, biosecurity, vaccination, brooding, housing, equipment, personnel, markets, extension, AMR, antimicrobial usage, mortality, and public health.

To create a meaningful order and format, the questions were rationally arranged, grouping related themes. To evaluate the questionnaire’s clarity, flow, question interpretation, and completion time, as well as to gather input on any unclear or problematic areas, a pilot was conducted with a small convenience sample of 5 respondents rearing small- to medium-scale poultry. Respondents’ cognitive effort and five-point difficulty rating upon completing the interview were assessed by the interviewer, who noted whenever a respondent hesitated, looked confused, or asked to have a question repeated, and by respondents, who rated how difficult it was to remember or answer specific questions on a scale of 1 to 5. Consequently, the pilot results showed an extended completion time of 2-3 h, necessitating optimization to ensure it was as short as possible while containing all pertinent information and striking a balance between comprehensiveness and respondent burden to maximize completion rates. Pilot results also revealed that some respondents had difficulty remembering the precise dates of vaccinations and antibiotic purchases, and certain questions about biosecurity measures were interpreted inconsistently (e.g., footbath presence vs. proper use). Based on this feedback, compound biosecurity questions were split into distinct items, and some open-ended variables were substituted with categorical options. The flow was generally enhanced by these modifications, which also helped to reduce completion time in further interviews. Data enrolment was done directly by means of Epi-Collect 5^1^ and Excel spreadsheets.

The objectives were to develop a tool that, when used independently at the farm level, could assist

1. In monitoring the performance of variable domain farm factors and benchmarking farms as performing above or below average.
2. In evaluating the relationship between variable domain factors.
3. In assessing the distribution of AMU by farm factors, e.g., if used in combination with AMU surveillance systems such as the Veterinary Consumption of Antibiotics—International Documentation (VetCAb-ID) system (©TiHo Hannover, Germany) (41, 43).
4. In evaluating the relationships between antimicrobial usage and farm factors.

### 2.2 Data collection

The primary questionnaire was used to collect original raw data from small- to medium-sized broiler poultry farms (100 to 5,000 birds) between 2021 and 2023 across seasonal months as described in detail in our previously published longitudinal antimicrobial use work (41). In brief, the snowball sampling approach, in which new units recruit other units to form part of the sample, was used to recruit the participating farms. Initially, 10 farms were identified through local veterinary and marketplace contacts. Each of the initially selected farms recruited 1 to 4 additional farms, resulting in 45 farms enrolled after verbal consent. These farms largely operated as intensive, non- to semi-commercial farms and focused typically on local markets. At each farm, the questionnaire (combined paper-based and electronic) was administered to poultry owners, managers, or supervisors through in-person interview visits to all farms at the time of recruitment. The first author collected all data and performed domain mapping and variable scoring into Microsoft Excel before further processing (see Supplementary file 1). Lusaka was purposively selected to represent typical small-to-medium poultry practices in Zambia due to its capital status and dominance in trading poultry products. Enrolled farms were located across urban and peri-urban/rural areas of Lusaka district, with representation from high-density, medium-density, and low-density settlements. The sample size was an important factor because the collected data were to be used for the development and downstream analysis, utilizing statistical analyses to evaluate the tool’s validity and applicability, and to show first trends in statistical significance for the target population to which the study was addressed. A generally feasible sample size of *n* = 46 farms was recognized. For the general question of differences in treatment frequency (TF) (see Equation 1) (44), a Wilcoxon test was used. Assuming a statistical significance level of *α* = 0.05, a difference Δ = 2, and a general standard deviation of *σ* = 10, the expected power was calculated as (1 − *β*) = 0.763 for a log-normally distributed TF between groups within NCSS-PASS, version 2022. This was assumed sufficient power for an explorative observational study.

### 2.3 Statistical analysis for the development of the tool

A binary scoring system was incorporated into the tool’s design to evaluate farmer responses pertaining to positively coded data. Specifically, “1” as a response meant “Yes,” meaning that the farmer followed the best practices in the relevant domain, receiving 1 point. Conversely, a response of “0” indicated a “No,” indicating that the farmer did not adhere to the recommended best practices and was not awarded any points.

Variables with no variation, i.e., those with the same value for >42 farms, were eliminated from further analyses, as such variables do not contribute any additional useful information for analysis and therefore can potentially skew the results.

Afterwards, each variable was analyzed for association using Cramér’s *V* statistic, a measure of association between variables, ranging from 0 (no association) to 1 (perfect association). However, a value greater than 0.3 was considered an indication that there is an enlarged association between the variables, meaning that both variables have a similar explanatory value (45). Where 2 variables had a Cramér’s *V* of more than 0.3, we kept the variable that was deemed more directly related to IPC or AMU based on established associations in the AMR/AMU literature, biological plausibility, and actionability for stewardship interventions. The second variable of each pair of factors was eliminated as a redundant proxy. Consequently, variable reduction was performed in a two-step process: (1) elimination of variables using Cramér’s *V* pairwise association screening; (2) based on relevance for AMU/IPC as described above, 1 variable was eliminated for every combination. To maintain domain coverage while lowering redundancy, this procedure was used within each of the 13 domains. To evaluate the strength of the relationship, heatmaps for the Cramér’s *V* results were used. We have included an asterisk (*) for factors that were excluded. These analyses were investigated using Proc Freq and Proc Sgplot within SAS 9.4 TS level 1M7 (SAS Institute Inc., Cary, NC, United States).

### 2.4 Statistical analysis for the application of the tool

The application of the tool involved performing several analyses using statistical methods to assess the trends and patterns of the collected farm-level factors within poultry farms and their relationship to antimicrobial usage, which was documented via the VetCAb-ID data source as therapy frequency (44), i.e.,

$$\begin{array}{l} \mathrm{TF}\;\;[\text{treatment frequency}]=\\ \frac{\sum \text{treatments}\;(\text{animals treated}\times \text{treatment days})}{\text{animals in the population}} \end{array}$$ (1)

This general calculation (Equation 1) was performed per run and day and finally averaged over all runs per farm.

The relationship between farm factors and epidemiological factors on the one hand and treatment frequency on the other was screened with two-sample Wilcoxon tests at the single-factor level. These analyses were investigated at a 5% significance level using Proc NPAR1WAY within SAS 9.4 TS level 1M7 (SAS Institute Inc., Cary, NC, United States).

## 3 Results

### 3.1 Basic demographics on participating farmers and their farms

Overall, 45 farms in the district of Lusaka, Zambia, were enrolled for this project. Among the enrolled farmers, men and women were similarly represented (53.3% vs. 46.7%), and the median age of the farmers was 32 (range: 20 to 75 years). The majority (57.8%) of farms enrolled were run by single (unmarried) individuals, and almost half (51.1%) of farm owners had tertiary education. The majority of respondents were not formally employed (71.1%). The majority of the farms enrolled were from urban areas (75.6%), with fewer (24.4%) in peri-urban and rural areas. In terms of the farm role, the majority (82.2%) of those enrolled were farm owners. The median duration of poultry farming experience was 5 years (range: 4 months to 15 years). The median number of batches produced was 30, and the majority of farmers had produced between 1 and 20 batches annually. The median number of chickens reared by farmers enrolled was 400 (range: 100 to 5,000). The majority (66.7%) of farmers raised chickens for 42 to 49 days. The median cropping age was 42 days, with a minimum of 35 days and a maximum of 56 days. Most farmers (73.3%) used their own poultry houses, but a few rented (26.7%). Most farmers (57.8%) sold their chickens both live and dressed. Only a small proportion (4.4%) of farmers sold their chickens as dressed only, and 37.8% sold them as live only.

### 3.2 Results for the development of the tool

From the 175 factors, 32 were excluded from the analysis due to little to no variance (Supplementary file 1, Supplementary Table 3). The relationships between the 143 variables were tested using Cramér’s *V*. Variables with a Cramér’s *V* larger than 0.3 were investigated, and 1 of each pair was excluded based on relevance for AMU. Analysis was performed for each domain separately. For a better overview of each domain and the strength of the relationship between factors, heatmaps were used (Supplementary file 1, Supplementary Figures 1–12). From 143 variables, 42 variables were excluded due to strong relationships with other factors. Therefore, 101 variables were left after this analysis step. This resulted in a finalized tool (Supplementary file 2). Supplementary file 2 provides an Excel sheet to use as a template for data collection of all 101 variables.

### 3.3 Distribution of treatment frequencies by demographical factors

Overall, unemployed farmers had a statistically significantly higher treatment frequency compared to employed farmers. Farms that sold only live chickens also had a higher treatment frequency (*p* = 0.0014, Table 1). For all other demographic factors, no significant differences in treatment frequencies were detected.

**Table 1:** Treatment frequency by demographic factors.

Demographic factors		Mean TF per day									*p*-value^*^
		*n*	Mean	Median	STD	CV	Min	5%-quantile	95%-quantile	Max	
Employment status	Employed	13	0.10	0.09	0.09	86.92	0.00	0.00	0.33	0.33	0.0492
	Unemployed	32	0.19	0.14	0.16	83.62	0.00	0.04	0.47	0.83	
Sells chickens as	Both live and dressed	26	0.11	0.09	0.09	81.65	0.00	0.00	0.28	0.37	0.0014
	Dressed only	2	0.31	0.31	0.03	10.88	0.29	0.29	0.33	0.33	
	Live only	17	0.24	0.16	0.19	78.95	0.10	0.10	0.83	0.83	

^*^p-values of the two-sample Wilcoxon test for statistically significant results only. TF, treatment frequency; STD, standard deviation; CV, coefficient of variation; Min, minimum; Max, maximum.

### 3.4 Distribution of treatment frequencies by epidemiological factors

An analysis of the distribution of the treatment frequency for the validated variables (*N* = 101) was performed. To determine if the distribution of treatment frequency varied across farms that adhered to each binary farm-factor indicator and those that did not, two-sample Wilcoxon tests were used to characterize an association between an epidemiological factor and antimicrobial use. Six variables showed a statistically significant relationship with TF. The results show that supplementation with vitamins, vitamins prior to vaccination, the use of measures to enhance chick survival (such as vitamins, prophylactics, or sugar), adequate litter thickness, inadequate number of drinkers for older chickens, and the farmers’ belief that maximum production cannot be achieved without antibiotics all increased the TF. For vitamin supplementation, measures of survival, and number of drinkers, less than 9% of farms did not adhere to the recommended best practices. For litter depth, 15.5% followed best practices (Table 2).

**Table 2:** Treatment frequency by epidemiological factors.

Epidemiological factors		Mean TF per day									*p*-value^*^
		*n*	Mean	Median	STD	CV	Min	5%-quantile	95%-quantile	Max	
Vitamin supplementation	No	4	0.06	0.07	0.05	81.3	0.00	0.00	0.10	0.10	0.0328
	Yes	41	0.18	0.14	0.15	85.2	0.00	0.03	0.38	0.83	
Vitamins administration pre-vaccination	No	19	0.11	0.10	0.09	81.4	0.00	0.00	0.37	0.37	0.0094
	Yes	26	0.21	0.16	0.17	81.2	0.00	0.04	0.47	0.83	
Measures for survival	No	3	0.05	0.06	0.04	90.4	0.00	0.00	0.08	0.08	0.0324
	Yes	42	0.18	0.13	0.15	85.4	0.00	0.03	0.38	0.83	
Litter depth for chickens	No	38	0.14	0.12	0.10	72.5	0.00	0.00	0.33	0.38	0.0345
	Yes	7	0.32	0.18	0.27	83.7	0.11	0.11	0.83	0.83	
Number of drinkers for older chickens	No	3	0.33	0.33	0.06	17.3	0.27	0.27	0.38	0.38	0.0242
	Yes	42	0.16	0.12	0.15	94.6	0.00	0.00	0.37	0.83	
Maximum production without antibiotics	No	28	0.20	0.14	0.17	83.1	0.03	0.05	0.47	0.83	0.0393
	Yes	17	0.11	0.10	0.09	83.1	0.00	0.00	0.33	0.33	

^*^p-values of the two-sample Wilcoxon test for statistically significant results only. TF, treatment frequency; STD, standard deviation; CV, coefficient of variation; Min, minimum; Max, maximum.

## 4 Discussion

In this study, we report the development and application of an epidemiological tool, EpiF*AMU*, that can monitor farm-level factors essential to AMU and IPC programs in poultry production systems against the background of a production system in Africa. At the farm level, the tool can monitor multiple epidemiological variables in 13 critical areas of poultry management and systems. The development of the tool shows that, ultimately, 101 variables are sufficient to operate the tool. The final version of the tool is now accessible for use as an electronic or paper-based system (Supplementary file 2). The tool can be used either independently to monitor farm-level variables or in conjunction with other systems, such as the VetCAb-ID (©TiHo Hannover, Germany), to monitor both antimicrobial use and farm factors at the farm level.

The tool includes a basic counting score for each domain to compare farms directly. These scores can be used to benchmark farms as performing above or below average. This benchmarking system needs to be evaluated in future studies, especially the use of these scores in relation to AMU. The tool generally compares with existing programs such as Biocheck UGent (62). However, the UGent system offers a risk-weighted biosecurity score for several livestock species, including poultry, in which the weighting procedures are mainly driven by the European perspective. Therefore, EpiF*AMU* provides a more comprehensive epidemiological profile of farm-level determinants by covering 13 domains from an African perspective.

### 4.1 Treatment frequencies by demographic and epidemiological factors

Generally, we assessed the patterns and relationships between farm factors and antimicrobial usage. Farmers who believed that maximum production cannot be achieved without antibiotics had a significantly higher treatment frequency compared to those who did not. This finding is supported by other studies, which reported that farmers who believe that using antimicrobials is necessary to achieve maximum productivity typically use them more frequently (46). The behavioral, social, and cultural factors may influence the use of antimicrobials (38, 47), and therefore these attributes of the habitual use of antimicrobials are potentially mediated by behavioral aspects (8, 47). This suggests the necessity of focused interventions to change attitudes and enhance antimicrobial stewardship among poultry farmers. Evidence from countries with stringent regulations and high levels of farmer awareness demonstrates that optimizing production may be achieved without the regular use of antimicrobials, suggesting that antimicrobial use can be significantly reduced without compromising production through targeted policy, education, and alternative management practices (48).

Farmers who did not provide an adequate number of drinkers (i.e., water dispensing points) for older chickens had a significantly higher treatment frequency compared to farmers who did. An adequate number of drinkers is needed, particularly in the hot season when the birds become stressed by heat and require access to water to cool down. In this study, an adequate number of drinkers was defined as having at least 1 bell drinker per 100 birds. Therefore, not having access to water can stress the birds, and the farmer could be tempted to treat. However, there is no adequate evidence that highlights the relationship between the number of drinkers in older chickens and the level of antimicrobial usage. Some evidence available suggests that mass medication through drinking water is the primary method of administering antimicrobials to poultry and that this practice is associated with increased antimicrobial usage and resistance (49). Although the scale of mass medication, which generally rises with flock size and age, is used to infer the relationship, further research would be required to understand a direct quantitative relationship.

Unemployed farmers or farmers not engaged in other income-generating activities had a significantly higher treatment frequency compared to employed farmers. This could be due to the fact that raising chickens is frequently the primary source of income for unemployed people, which increases their dependence on optimizing flock survival and productivity. It could also mean they have little income to invest in hygiene measures and thus depend on treatment to compensate for hygiene failures. They may also not have resources to pay for veterinary services. Other studies have reported that increased and more frequent use of antimicrobials, often without veterinary consultation or adherence to appropriate withdrawal periods, can be attributed to this economic pressure (50–52).

Farmers who sold chickens both live and dressed had a significantly higher treatment frequency compared to those who sold them live only or dressed only. There are equally limited data comparing the levels of antimicrobial usage by farmers who sell dressed versus live chickens. Nonetheless, it is quite possible that going to the market could easily result in a breach of biosecurity as farmers are most likely to bring infections from the market, resulting in the use of antimicrobials. In both situations, however, some evidence suggests that antimicrobials are frequently used until the point of sale, particularly in settings with weak regulation (36, 50, 53).

In contrast, some “good” farming practices that would ideally be expected to decrease AMU were linked to an increase in AMU. For instance, farmers who supplemented chickens with vitamins had a significantly higher treatment frequency compared to those who did not use them. This was contrary, as evidence suggests that farmer practices that include adding vitamins to chicken diets are linked to lower levels of antimicrobial usage primarily mediated by improved general health and immune function, which reduces the incidence of diseases that typically require the use of antimicrobials (54, 55). Similarly, farmers who provided chickens with vitamins prior to vaccination had a significantly higher treatment frequency compared to those who did not. This was also contrary, as evidence suggests that administering vitamins to chickens prior to vaccinations boosts their immune systems and may minimize the incidence of diseases, which could reduce the need for antimicrobials (54, 56). Further, farmers who used measures to enhance chick survival (vitamins, prophylactics, or sugar) had a significantly higher treatment frequency compared to those who did not use them. This was equally contrary because evidence suggests that farmers are more likely to rely on antimicrobials when they do not use alternative health management strategies to increase chick survival (53, 57). The reason for the association between practices such as vitamin usage and high treatment frequency is not apparent, but may be associated with high antimicrobial usage in poultry because some commercial vitamin products also include antibiotics, and this practice is especially prevalent in regions with insufficient regulation of veterinary medicines (58). However, it is certainly conceivable that the group under investigation is subject to an information bias here as no precise definition of “vitamins” and “antimicrobials” had been explicitly provided during this study. Therefore, we suspect that some farmers may have confused vitamins with antimicrobials, which may explain the higher treatment frequency in farms with vitamin use.

Farmers who provided an adequate thickness of litter had a significantly higher treatment frequency compared to farmers who did not. This was also contrary because evidence suggests that poor litter management increases the risk of disease in chickens, which leads to a rise in the need for treatment (8, 57, 59). It is, however, possible that thicker litter is likely to protect pathogens and maintain their survival in the environment. These counterintuitive patterns have also been reported in other studies. For example, a study in Fijian livestock farms found that keeping records (a good practice) was associated with higher antimicrobial usage (60). Such counterintuitive patterns could arise from several factors. It might imply that farms with greater problems, such as more disease pressure, are more likely to implement recommended “good” procedures; however, because of their increased baseline risk, they also use more antimicrobials despite these measures. Conversely, even though their practices are objectively “bad,” farms with fewer problems or less disease pressure may not adopt “good” practices (because they perceive less need) and appear to use fewer antimicrobials (61). However, direct studies specifically measuring the relationship of such counterintuitive patterns in poultry production systems remain an area for further research.

### 4.2 Limitations

Limitations associated with the data used in this study were extensively discussed in our earlier published work (41). Briefly, data collection was limited by practical and financial factors, and some limitations were noted. In the absence of population statistics and broiler poultry farm registries, it was not possible to apply a probability-based random sampling technique. Therefore, the study is prone to a selection bias, which in addition may be responsible for some of the contrary results stated above. Additionally, snowball sampling itself may have contributed to a sample of farms with similar management habits, which may also contribute to our contrary results. Also, some farms did not want to participate in this study as they did not want to share their information on AMU. This sampling system and the absence of poultry farm registries lead to a low sample size of 45 farms compared to 101 analyzed variables. Accordingly, caution must be taken when generalizing the results of this exploratory investigation.

Data collection was performed via an in-person questionnaire. Since the questions were based on whether or not a farm adhered to the recommended best practices, answers may be prone to a social desirability bias, and therefore farms with bad practices may have under-reported. Additionally, recall bias may have been introduced for some questions.

For the relationship between epidemiological and demographic factors and TF, no adjustment for multiple testing was used, although we tested over 100 different variables. Since we consider our study exploratory, we did not adjust for multiple testing and are cautious in interpreting these results since some of these results may have occurred by chance. However, it is interesting to us that even without correction, only 8 variables were identified within this exploration. This highlights the need for a study in which the tool developed is tested on a different and larger sample. Especially considering the low sample size in some groups, results should be interpreted with caution.

Furthermore, awareness of AMR was the only variable assessed in the AMR domain of this tool. This reflects the study’s primary focus on farm factors influencing AMU and the practical limitation that the majority of small-to-medium farms in this study setting do not perform routine on-farm AMR testing. Therefore, this study was unable to measure direct AMR outcomes at the farm level. Future studies should include objective AMR indicators such as periodic fecal or litter sampling with culture or molecular identification of resistant profiles. As a capital city with a high level of poultry activity, Lusaka was chosen as a typical representation. The sample was restricted to small- to medium-sized broiler farms in Lusaka district and may not represent other poultry production systems or regions in Zambia.

Beyond these limitations, our findings are relevant and demonstrate the feasibility of monitoring farm factors to generate meaningful insights into possible risk factors within broiler poultry farms that may lead to the use of antimicrobials. The developed tool provides a simple and ready-to-use solution that enables the systematic collection of important farm factor data, facilitating benchmarking of metrics at the farm, country, or regional levels in terms of antimicrobial usage and farm factors.

## 5 Conclusion

EpiF*AMU* provides a practical, low-cost solution for systematic monitoring of farm-level factors relevant to AMU and IPC in poultry systems. The tool can be used to track management practices linked to increased treatment frequencies, benchmark farms against domain-specific averages, and guide focused antimicrobial stewardship and infection prevention programs at the farm, district, and national levels. Future research should aim to decipher EpiF*AMU*’s predictive performance for AMU and AMR outcomes, validating it externally in larger, stratified samples across geographical locations and livestock systems, and conducting a longitudinal analysis to assess whether changes in EpiF*AMU* scores over time correlate with reduced antimicrobial use and AMR.

## Data Availability

The data were collected on an individual basis from farmers. Each participant provided consent with the understanding that data would not be transferred to any third party. Therefore, data transfer to interested persons is not allowed without an additional formal contract. Data are available to qualified researchers who sign a contract with University of Zambia (UNZA) and University of Veterinary Medicine Hannover (TiHo). This contract will include guarantees of the obligation to maintain data confidentiality in accordance with the provisions of the University of Zambia and the European General Data Protection Regulation and its supporting documents in Germany. Currently, there is no data access committee or another body who could be contacted for the data. However, for this purpose, a committee will be founded. This future committee will consist of the authors, as well as other members of UNZA and TiHo. Interested cooperative partners who are able to sign a contract as described above may contact the corresponding author or authors affiliated to the University of Zambia. Requests to access the datasets should be directed to Lothar Kreienbrock (lothar.kreienbrock@tiho-hannover.de) or Isaac Silwamba (isaacsilwambak@gmail.com).

## Footnotes

1
https://five.epicollect.net/
