# Supplementary material for: EpiFAMU: an epidemiological tool for monitoring farm-level factors essential to antimicrobial use surveillance and infection prevention and control programs in poultry production systems in Zambia—development and application
Source: Front Vet Sci. 2026 Oct 22;13:1963471. doi: 10.3389/fvets.2026.1963471 (PMC13735731; doi:10.3389/fvets.2026.1963471)
Supplement: Supplementary file 1 [file Data_Sheet_2.DOCX]

Supplementary Material

Supplementary Table 1: Description of treatment frequencies per day and per day and livestock

|  | Mean TF | | | | | | | | |
| --- | --- | --- | --- | --- | --- | --- | --- | --- | --- |
|  | n | mean | median | STD | CV | min | 5%-quantile | 95%-quantile | max |
| **per day** | 135 | 0.17 | 0.14 | 0.16 | 94.15 | 0.00 | 0.00 | 0.43 | 0.83 |
| **per day and livestock** | 45 | 0.17 | 0.12 | 0.15 | 89.16 | 0.00 | 0.00 | 0.38 | 0.83 |

Abbreviations: TF = treatment frequency; STD = standard deviation; CV = coefficient of variation; min = minimum, max = maximum

Supplementary Table 2: Treatment frequency per day for each livestock

| livestock | N | Min | Mean | Median | Max |
| --- | --- | --- | --- | --- | --- |
| 14 | 3 | 0.00000 | 0.00000 | 0.00000 | 0.00000 |
| 22 | 3 | 0.00000 | 0.00000 | 0.00000 | 0.00000 |
| 30 | 3 | 0.00000 | 0.00000 | 0.00000 | 0.00000 |
| 40 | 3 | 0.00000 | 0.03175 | 0.00000 | 0.09524 |
| 7 | 3 | 0.00000 | 0.03968 | 0.00000 | 0.11905 |
| 34 | 3 | 0.00000 | 0.03968 | 0.00000 | 0.11905 |
| 39 | 3 | 0.00000 | 0.04762 | 0.07143 | 0.07143 |
| 8 | 3 | 0.00000 | 0.05587 | 0.06122 | 0.10638 |
| 26 | 3 | 0.00000 | 0.06803 | 0.00000 | 0.20408 |
| 21 | 3 | 0.00000 | 0.06973 | 0.10204 | 0.10714 |
| 28 | 3 | 0.00000 | 0.07937 | 0.11905 | 0.11905 |
| 32 | 3 | 0.00000 | 0.07937 | 0.00000 | 0.23810 |
| 25 | 3 | 0.06122 | 0.08844 | 0.10204 | 0.10204 |
| 5 | 3 | 0.00000 | 0.09524 | 0.14286 | 0.14286 |
| 33 | 3 | 0.00000 | 0.09524 | 0.14286 | 0.14286 |
| 4 | 3 | 0.05357 | 0.09949 | 0.12245 | 0.12245 |
| 1 | 3 | 0.00000 | 0.10110 | 0.06522 | 0.23810 |
| 18 | 3 | 0.08929 | 0.10714 | 0.08929 | 0.14286 |
| 27 | 3 | 0.06122 | 0.10884 | 0.12245 | 0.14286 |
| 15 | 3 | 0.09524 | 0.11111 | 0.09524 | 0.14286 |
| 16 | 3 | 0.08929 | 0.11310 | 0.12500 | 0.12500 |
| 37 | 3 | 0.11905 | 0.11905 | 0.11905 | 0.11905 |
| 29 | 3 | 0.00000 | 0.11905 | 0.17857 | 0.17857 |
| 19 | 3 | 0.07143 | 0.12230 | 0.08333 | 0.21214 |
| 38 | 3 | 0.14286 | 0.14286 | 0.14286 | 0.14286 |
| 45 | 3 | 0.14286 | 0.14286 | 0.14286 | 0.14286 |
| 2 | 3 | 0.12727 | 0.15403 | 0.15114 | 0.18367 |
| 6 | 3 | 0.13462 | 0.15598 | 0.16667 | 0.16667 |
| 10 | 3 | 0.06122 | 0.16327 | 0.16327 | 0.26531 |
| 24 | 3 | 0.14286 | 0.16327 | 0.14286 | 0.20408 |
| 44 | 3 | 0.16667 | 0.16667 | 0.16667 | 0.16667 |
| 41 | 3 | 0.14286 | 0.17687 | 0.14286 | 0.24490 |
| 17 | 3 | 0.07143 | 0.18254 | 0.11905 | 0.35714 |
| 3 | 3 | 0.11905 | 0.19841 | 0.23810 | 0.23810 |
| 23 | 3 | 0.16327 | 0.26531 | 0.16327 | 0.46939 |
| 20 | 3 | 0.26786 | 0.26786 | 0.26786 | 0.26786 |
| 31 | 3 | 0.28095 | 0.28413 | 0.28571 | 0.28571 |
| 11 | 3 | 0.28571 | 0.28571 | 0.28571 | 0.28571 |
| 36 | 3 | 0.33333 | 0.33333 | 0.33333 | 0.33333 |
| 42 | 3 | 0.33333 | 0.33333 | 0.33333 | 0.33333 |
| 43 | 3 | 0.33333 | 0.33333 | 0.33333 | 0.33333 |
| 12 | 3 | 0.36735 | 0.36972 | 0.36735 | 0.37447 |
| 35 | 3 | 0.33333 | 0.38095 | 0.33333 | 0.47619 |
| 13 | 3 | 0.42857 | 0.46939 | 0.42857 | 0.55102 |
| 9 | 3 | 0.83333 | 0.83333 | 0.83333 | 0.83333 |

Abbreviations: TF = treatment frequency; min = minimum, max = maximum

Supplementary Table 3: Distribution of answers for each epidemiological question per domain

|  | | | Response | |
| --- | --- | --- | --- | --- |
|  |  |  | no | yes |
| Domain | Question no | Question |  |  |
| Nutrition | 1 | Type of feed | 1 | 44 |
|  | 2 | Source of feed | 1 | 44 |
|  | 3 | Feed quality monitoring | 44 | 1 |
|  | 4 | Adlib feeding | 30 | 15 |
|  | 5 | Drinking water | . | 45 |
|  | 6 | Feed spilage | 41 | 4 |
|  | 7 | Vitamin supplementation | 4 | 41 |
|  | 8 | Feeding other apart feeds | 21 | 24 |
|  | 9 | Feeding program manipulation | 40 | 5 |
|  | 10 | Cropping age | 6 | 39 |
|  | 11 | Weight monitoring | 29 | 16 |
| Veterinary | 1 | Encounter disease | 45 | . |
|  | 2 | Method of managing disease | 35 | 10 |
|  | 3 | First 2 Weeks Growth period disease occurrence | 29 | 16 |
|  | 4 | Week 5 Growth period disease occurrence | 9 | 36 |
|  | 5 | Regular poultry health program by vet | 45 | . |
|  | 6 | How much lighting | 38 | 7 |
|  | 7 | Deworm chickens | 44 | 1 |
|  | 8 | Mortality rate | 26 | 19 |
|  | 9 | Period year mortalities | 4 | 41 |
|  | 10 | Detection of non healthy birds & Clinical signs | . | 45 |
|  | 11 | Flock inspection | 38 | 7 |
|  | 12 | Chick problems hatchery & farm | 3 | 42 |
| Biosecurity | 1 | Housed chickens | . | 45 |
|  | 2 | Production cycle | 18 | 27 |
|  | 3 | Drainage discharge point | 44 | 1 |
|  | 4 | Mix different bird type | 8 | 37 |
|  | 5 | Mix different age group | 4 | 41 |
|  | 6 | Animals near housing | 32 | 13 |
|  | 7 | Housing fenced off | 37 | 8 |
|  | 8 | Housing restricted | 33 | 12 |
|  | 9 | Activities before bringing chickens | 1 | 44 |
|  | 10 | Protective clothing | 22 | 23 |
|  | 11 | Wash hands between visits | 24 | 21 |
|  | 12 | Hygienic practices consitency | 34 | 11 |
|  | 13 | Pests and rodent control program | 36 | 9 |
|  | 14 | Isolation facility | 39 | 6 |
|  | 15 | Foot bath | 14 | 31 |
|  | 16 | Chemical filling in foot bath | 29 | 16 |
|  | 17 | Shed on foot bath | 37 | 8 |
|  | 18 | Workers limit visitations | 30 | 15 |
|  | 19 | Areas and storage clean | 5 | 40 |
|  | 20 | Disifect equipment | 9 | 36 |
|  | 21 | Equipment exchange | 2 | 43 |
|  | 22 | Vehicle coming in yard | 43 | 2 |
|  | 23 | Exclusive foot wear | 8 | 37 |
|  | 24 | Pets in poultry house | 7 | 38 |
|  | 25 | Wear protective clothing visitors | 39 | 6 |
|  | 26 | Isolate broilers from other poultry | 23 | 22 |
|  | 27 | Treat chlorine water | 33 | 12 |
|  | 28 | Sweep poultry house | . | 45 |
|  | 29 | Apply detergent | 19 | 26 |
|  | 30 | Waiting between batches | 17 | 28 |
|  | 31 | Influence waiting | 34 | 11 |
|  | 32 | Cleaning chemicals | 10 | 35 |
|  | 33 | Who provide support calculating | 38 | 7 |
|  | 34 | Recycle bedding | 3 | 42 |
|  | 35 | Wet liiter | 43 | 2 |
|  | 36 | Manage wet litter | 8 | 37 |
|  | 37 | Prevent wet liiter | 29 | 16 |
|  | 38 | Disposal of used litter storage | 16 | 29 |
|  | 39 | Feed storage | 23 | 22 |
|  | 40 | Water quality | 44 | 1 |
|  | 41 | Source of water | 3 | 42 |
|  | 42 | Mortality chicken disposal practices burry, throw | 36 | 9 |
|  | 43 | Duration before disposal | 4 | 41 |
|  | 44 | Feeding of mortality chickens to pets | 28 | 17 |
| Vaccination | 1 | Regurlary vaccinate | . | 45 |
|  | 2 | What vaccines | . | 45 |
|  | 3 | What schedule | 34 | 11 |
|  | 4 | Health before vaccination | 3 | 42 |
|  | 5 | Vitamines pre vaccination | 19 | 26 |
|  | 6 | Vitamins post vaccination | 16 | 29 |
|  | 7 | Water non chlorinated | 10 | 35 |
|  | 8 | Drinker not dinsifectens | 4 | 41 |
|  | 9 | Water boiled,aviblue or skimmed milk | 32 | 13 |
|  | 10 | Water cool | 4 | 41 |
|  | 11 | Open in water | 11 | 34 |
|  | 12 | Waiting before giving | 8 | 37 |
|  | 13 | Calculate volume of water | 37 | 8 |
|  | 14 | Water starvation | 29 | 16 |
|  | 15 | Distribut vaccine | 27 | 18 |
|  | 16 | Storage during transport | 38 | 7 |
|  | 17 | Home directly | 8 | 37 |
|  | 18 | Reach home cool and storage | . | 45 |
|  | 19 | Vaccnine storage | 2 | 43 |
|  | 20 | Method of administration | 1 | 44 |
|  | 21 | Duration for consumption | 36 | 9 |
|  | 22 | Expiry of vaccines | 6 | 39 |
| Brooding | 1 | Length of brooding | 16 | 29 |
|  | 2 | Source of birds | 23 | 22 |
|  | 3 | Stocking density chicks per square meter | 23 | 22 |
|  | 4 | Measures for survival | 3 | 42 |
|  | 5 | Type of bedding chicks | 12 | 33 |
|  | 6 | Litter depth chicks | 38 | 7 |
|  | 7 | Preheat house | 5 | 40 |
|  | 8 | When preheat | 40 | 5 |
|  | 9 | Feed and water available | 5 | 40 |
|  | 10 | Supplementary feeders | 11 | 34 |
|  | 11 | Period before unloading | 24 | 21 |
|  | 12 | Check feed, water, temp, hum | . | 45 |
|  | 13 | Interval for checking day time | 18 | 27 |
|  | 14 | Times waking up night/sleep in poultry | 25 | 20 |
|  | 15 | Monitor chick quality | 3 | 42 |
|  | 16 | Chick quality characteristcss | 37 | 8 |
|  | 17 | Mode of transport chicks | 10 | 35 |
|  | 18 | Transport period | 8 | 37 |
|  | 19 | Mode of heating | 43 | 2 |
|  | 20 | Number of heating in brooder | 26 | 19 |
|  | 21 | Heating method works better | 20 | 25 |
| Housing | 1 | Type of housing | 6 | 39 |
|  | 2 | Size of poultry house | 12 | 33 |
|  | 3 | Floor type | 7 | 38 |
|  | 4 | Chilling | 22 | 23 |
|  | 5 | Predators | 14 | 31 |
|  | 6 | Rain leakage | 13 | 32 |
|  | 7 | Ventilation | 10 | 35 |
|  | 8 | Roof overhang | 7 | 38 |
|  | 9 | Flooding | 18 | 27 |
|  | 10 | Orientation | 15 | 30 |
|  | 11 | Height | 21 | 24 |
|  | 12 | Side walls | 15 | 30 |
|  | 13 | Wire mesh | 4 | 41 |
|  | 14 | Drainage | 29 | 16 |
|  | 15 | Air quality | 45 | . |
|  | 16 | Humidity | 45 | . |
|  | 17 | Electricity/source of power | 1 | 44 |
|  | 18 | Temperature regulation in house | 45 | . |
| Equipment | 1 | Type feeders older | 35 | 10 |
|  | 2 | Type drinkkers older | 2 | 43 |
|  | 3 | Number of drinkers older | 3 | 42 |
|  | 4 | Number of feeders older | 9 | 36 |
|  | 5 | Type of feeders for chick | 8 | 37 |
|  | 6 | Type of drinkers for chick | 7 | 38 |
|  | 7 | Number of drinkers in brooder | 10 | 35 |
|  | 8 | Number of feeders in brooder | 4 | 41 |
|  | 9 | Thermometer | 45 | . |
| Personnel | 1 | Formal training in poultry | 37 | 8 |
|  | 2 | Where do you get information | 37 | 8 |
|  | 3 | Who helps running poultry | 36 | 9 |
|  | 4 | Helpers for poultry | 9 | 36 |
|  | 5 | Willing to train | 6 | 39 |
| Market | 1 | Challenge selling | 37 | 8 |
|  | 2 | Medicines affect taste | 13 | 32 |
|  | 3 | Medicine affect sales | 16 | 29 |
|  | 4 | Where do you sale | 18 | 27 |
|  | 5 | How do you makert | 25 | 20 |
|  | 6 | Scalling up | 5 | 40 |
|  | 7 | Record keeping | 8 | 37 |
| Extension | 1 | Acess to agrovets support | . | 45 |
|  | 2 | Whom do you prefer | 24 | 21 |
|  | 3 | Hatchery technical support | 36 | 9 |
| AMR | 0 | Are you aware of AMR | 31 | 14 |
| Mortality | 1 | Request to sale dead | 31 | 14 |
|  | 2 | Do they consume | . | 45 |
|  | 3 | Know if selling dead | 11 | 34 |
|  | 4 | Safe to consume | 12 | 33 |
|  | 5 | Mortality treatment | 40 | 5 |
| AMU | 1 | Prescription | 43 | 2 |
|  | 2 | Store antibiotics | 42 | 3 |
|  | 3 | Who administer | 40 | 5 |
|  | 4 | Withdraw period | 34 | 11 |
|  | 5 | Isolate treat sick | 34 | 11 |
|  | 6 | Stock antimicrobials home | 37 | 8 |
|  | 7 | Drugs not effective | 38 | 7 |
|  | 8 | Inadequate extension on amu | 18 | 27 |
|  | 9 | Antimicrobial prevent disease | 38 | 7 |
|  | 10 | Antimicrobials boost growth | 22 | 23 |
|  | 11 | Maximum production without | 28 | 17 |
|  | 12 | Is combination | 27 | 18 |
|  | 13 | Human and animals antimicrobials | 30 | 15 |
|  | 14 | Use indegenous | 31 | 14 |
|  | 15 | Why use them | 33 | 12 |
|  | 16 | Always accompanied by antibiotics | 29 | 16 |
|  | 17 | Improved management amu | 25 | 20 |

Abbreviations: AMR = antimicrobial resistance, AMU = antimicrobial use

**Supplementary Figure 1.** Heatmap for Cramér’s V results for the **nutrition** domain. 4 = Adlib feeding, 6 = Feed silage, 7 = Vitamin supplementation, 8 = Feeding other apart feeds, 9 = Feeding program manipulation, 10 = Cropping age, 11 = Weight monitoring. Factors marked with * were excluded from further analyses due to high association.

**Supplementary Figure 2.** Heatmap for Cramér’s V results for the **veterinary** domain. 2 = Method of managing disease, 3 = First 2 Weeks Growth period disease occurrence, 4 = Week 5 Growth period disease occurrence, 6 = How much lighting, 8 = Mortality rate, 9 = Period year mortalities, 11 = Flock inspection, 12 = Chick problems hatchery & farm. Factors marked with * were excluded from further analyses due to high association.

**Supplementary Figure 3.** Heatmap for Cramér’s V results for the **biosecurity** domain. 2 = Production cycle, 4 = Mix different bird type, 5 = Mix different age group, 6 = Animals near housing, 7 = Housing fenced off, 8 = Housing restricted, 10 = Protective clothing, 11 = Wash hands between visits, 12 = Hygienic practices consistency, 13 = Pests and rodent control program, 14 = Isolation facility, 15 = Foot bath, 16 = Chemical filling in foot bath, 17 = Shed on foot bath, 18 = Workers limit visitations, 19 = Areas and storage clean, 20 = Disinfect equipment, 23 = Exclusive foot wear, 24 = Pets in poultry house, 25 = Wear protective clothing visitors, 26 = Isolate broilers from other poultry, 27 = Treat chlorine water, 29 = Apply detergent, 30 = Waiting between batches, 31 = Influence waiting, 32 = Cleaning chemicals, 33 = Who provide support calculating, 34 = Recycle bedding, 36 = Manage wet litter, 37 = Prevent wet litter, 38 = Disposal of used litter storage, 39 = Feed storage, 41 = Source of water, 42 = Mortality chicken disposal practices burry, throw, 43 = Duration before disposal, 44 = Feeding of mortality chickens to pets. Factors marked with * were excluded from further analyses due to high association.

**Supplementary Figure 4.** Heatmap for Cramér’s V results for the **vaccination** domain. 3 = What schedule, 4 = Health before vaccination, 5 = Vitamins pre vaccination, 6 = Vitamins post vaccination, 7 = Water non chlorinated, 8 = Drinker not disinfectants, 9 = Water boiled, aviblue or skimmed milk, 10 = Water cool, 11 = Open in water, 12 = Waiting before giving, 13 = Calculate volume of water, 14 = Water starvation, 15 = Distribute vaccine, 16 = Storage during transport, 17 = Home directly, 21 = Duration for consumption, 22 = Expiry of vaccines. Factors marked with * were excluded from further analyses due to high association.. Factors marked with * were excluded from further analyses due to high association.

**Supplementary Figure 5.** Heatmap for Cramér’s V results for the **brooding** domain. 1 = Length of brooding, 2 = Source of birds, 3 = Stocking density chicks per square meter, 4 = Measures for survival, 5 = Type of bedding chicks, 6 = Litter depth chicks, 7 = Preheat house, 8 = When preheat, 9 = Feed and water available, 10 = Supplementary feeders, 11 = Period before unloading, 13 = Interval for checking day time, 14 = Times waking up night/sleep in poultry, 15 = Monitor chick quality, 16 = Chick quality characteristics, 17 = Mode of transport chicks, 18 = Transport period, 20 = Number of heating in brooder, 21 = Heating method works better. Factors marked with * were excluded from further analyses due to high association.

**Supplementary Figure 6.** Heatmap for Cramér’s V results for the **housing** domain. 1 = Type of housing, 2 = Size of poultry house, 3 = Floor type, 4 = Chilling, 5 = Predators, 6 = Rain leakage, 7 = Ventilation, 8 = Roof overhang, 9 = Flooding, 10 = Orientation, 11 = Height, 12 = Side walls, 13 = Wire mesh, 14 = Drainage. Factors marked with * were excluded from further analyses due to high association.

**Supplementary Figure 7.** Heatmap for Cramér’s V results for the **equipment** domain. 1 = Type feeders older, 3 = Number of drinkers older, 4 = Number of feeders older, 5 = Type of feeders for chick, 6 = Type of drinkers for chick, 7 = Number of drinkers in brooder, 8 = Number of feeders in brooder. Factors marked with * were excluded from further analyses due to high association.

**Supplementary Figure 8.** Heatmap for Cramér’s V results for the **Personnel** domain. 1 = Formal training in poultry, 2 = Where do you get information, 3 = Who helps running poultry, 4 = Helpers for poultry, 5 = Willing to train. Factors marked with * were excluded from further analyses due to high association.

**Supplementary Figure 9.** Heatmap for Cramér’s V results for the **market** domain. 1 = Challenge selling, 2 = Medicines affect taste, 3 = Medicine affect sales, 4 = Where do you sale, 5 = How do you makert, 6 = Scalling up, 7 = Record keeping. Factors marked with * were excluded from further analyses due to high association.

**Supplementary Figure 10.** Heatmap for Cramér’s V results for the **extension** domain. 2 = Whom do you prefer, 3 = Hatchery technical support. Factors marked with * were excluded from further analyses due to high association.

**Supplementary Figure 11.** Heatmap for Cramér’s V results for the **mortality** domain. 1 = Request to sale dead, 3 = Know if selling dead, 4 = Safe to consume, 5 = Mortality treatment. Factors marked with * were excluded from further analyses due to high association.

**Supplementary Figure 12.** Heatmap for Cramér’s V results for the **antimicrobial use** domain. 2 = Store antibiotics, 3 = Who administer, 4 = Withdraw period, 5 = Isolate treat sick, 6 = Stock antimicrobials home, 7 = Drugs not effective, 8 = Inadequate extension on amu, 9 = Antimicrobial prevent disease, 10 = Antimicrobials boost growth, 11 = Maximum production without, 12 = Is combination, 13 = Human and animals antimicrobials, 14 = Use indegenous, 15 = Why use them, 16 = Always accompanied by antibiotics, 17 = Improved management amu. Factors marked with * were excluded from further analyses due to high association.
